# Salp swarm-optimized machine learning models for predicting preoperative aortic rupture risk in acute type a aortic dissection patients

**DOI:** 10.3389/fphys.2025.1675853

**Published:** 2025-10-29

**Authors:** Haiyue Bao, Lijun Sun, GuanQing Cui, Shihao Cai, Weiliang Zheng, Hua Peng, Chenhui Yang

**Affiliations:** ^1^ National Institute for Data Science in Health and Medicine, School of Medicine, Xiamen University, Xiamen, China; ^2^ Department of Intensive Care Unit, Xiamen Cardiovascular Hospital of Xiamen University, School of Medicine, Xiamen University, Xiamen, China; ^3^ School of Informatics, Xiamen University, Xiamen, China; ^4^ Department of Cardiac Surgery, Xiamen Cardiovascular Hospital of Xiamen University, School of Medicine, Xiamen University, Xiamen, China; ^5^ Department of Cardiac Rehabilitation, Xiamen Cardiovascular Hospital of Xiamen University, School of Medicine, Xiamen University, Xiamen, China

**Keywords:** type A aortic dissection, rupture risk prediction, clinical decision support, salp swarm optimization algorithm, machine learning

## Abstract

Acute Type A aortic dissection (ATAAD) is characterized by acute onset and rapid progression, with aortic rupture due to dissection extension being the primary lethal mechanism. Timely identification of high-risk patients is critical for prioritizing surgical intervention to reduce rupture incidence. This study aimed to develop and validate an interpretable machine learning model to predict aortic rupture in ATAAD patients, thereby improving risk classification and supporting clinical decisions. Medical records of ATAAD patients from Xiamen Cardiovascular Hospital (January 2019–October 2024) were retrospectively analyzed. Predictors were screened via statistical significance (p < 0.05) using seven machine learning algorithms, with the Salp Swarm Optimization Algorithm (SSA) optimizing hyperparameters for Random Forest and XGBoost models. To address class imbalance (47 rupture cases, 6.1%), SMOTE was implemented for data augmentation. Model performance was evaluated by accuracy, F1-score, precision, ROC-AUC, sensitivity, and specificity, supplemented by interpretability analyses through feature importance ranking and SHAP. Among 774 included ATAAD patients, the SSA-optimized Random Forest model achieved optimal performance (test dataset: 97.41% accuracy, 0.980 ROC-AUC, 81.82% F1-score). Key predictors included estimated glomerular filtration rate (eGFR), hypotension at admission, and white blood cell count. This work provides a quantitative tool for emergency care prioritization, with SSA enhancing model precision for high-risk patient identification, though multicenter studies are needed to validate generalizability.

## 1 Introduction

Aortic dissection (AD) is a life-threatening cardiovascular emergency characterized by a tear in the aortic intima-media layer, allowing high-pressure blood flow to penetrate the medial layer and propagate along the aortic axis, thereby creating true and false lumens (Nienaber and Clough, 2015; Schussnig et al., 2021). According to the Stanford classification, AD is categorized based on involvement of the ascending aorta: Type A dissections involve the ascending aorta and require emergency open surgical repair, typically involving ascending aortic replacement with or without hemiarch/total arch replacement (e.g., Sun’s procedure) (Zhu et al., 2020). Acute Type A aortic dissection (ATAAD), defined as occurring within 2 weeks of onset, is particularly critical due to its rapid progression. Mortality rates escalate by 1%–2% per hour post-onset, reaching 50% within 48 h Pichert et al. (2020), Pape et al. (2015), with studies reporting 24-h mortality as high as 47% rising to 55% at 48 h in untreated patients (Larsson et al., 2024). The primary causes of death include aortic rupture and malperfusion syndrome due to compromised blood supply to vital organs (Pichert et al., 2020). In China, the annual incidence is 2.78 per 100,000 population with a mean onset age of 51.6 years, showing a concerning trend toward younger demographics (Zhao et al., 2022). Early identification of high-risk rupture patients is therefore critical for improving survival.

Although some studies have explored preoperative rupture risk factors in ATAAD patients, significant limitations persist: (1) Small sample sizes (typically n<500 ) increase susceptibility to type II statistical errors; (2) Overreliance on anatomic imaging parameters (e.g., aortic diameter); with insufficient incorporation of serological biomarkers; (3) Limited focus on preoperative risk prediction models (Wu et al., 2019; Lin et al., 2023).

Recent advances in machine learning offer new opportunities to address these gaps. While random forest (RF) excels in handling nonlinear relationships Zhou et al. (2024), neural networks (NN) provide superior pattern recognition in complex datasets (Ghorrati et al., 2024). This study systematically evaluates machine learning algorithms (including RF, NN, and others) for ATAAD rupture prediction, leveraging both statistical robustness and clinical transparency to optimize surgical triage decisions.

## 2 Materials and methods

### 2.1 Study design, patients

**FIGURE 1:**
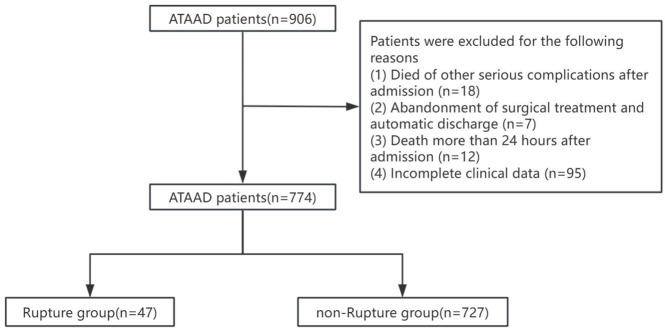
An overview of the flow through the study.

This study was a retrospective study. The research plan has been approved by the Ethics Committee of Xiamen Cardiovascular Hospital of Xiamen University (approved number: *KY2025-037*). Due to its retrospective design and anonymous nature, the requirement for patient informed consent is waived. Screening was conducted on patients diagnosed with ATAAD at Xiamen University Affiliated Cardiovascular Hospital from 1 January 2019 to 31 October 2024. Inclusion criteria: (1) Age ≥ 18 years, (2) onset time ≤ 14 days, (3) CTA diagnosis of ATAAD. Exclusion criteria: (1) Died of other serious complications after admission,(2) Abandonment of surgical treatment and automatic discharge, (3) Death more than 24 h after admission, (4) Incomplete clinical data. Figure 1 provides an overview of the research process. Ultimately, 774 eligible patients were included in the final analysis.

### 2.2 Data collection and processing

Clinical variables were collected at the time of patient admission, including demographic characteristics, clinical symptoms, laboratory biochemical tests, and ultrasound results. Laboratory variables (e.g., WBC, eGFR, and D-dimer) had missing values, with overall missingness less than 30% for any variable. Missing data were imputed using multiple imputation by chained equations (MICE) to reduce potential bias and maintain statistical power.

In order to prevent information leakage, all data preprocessing steps–including feature selection, standardization, and resampling with SMOTE–were strictly performed within the training and validation sets during model development, while the independent test set remained untouched throughout the model construction and Salp Swarm Optimization (SSA) tuning process. The dataset, comprising 774 samples in total, was randomly divided into training (n = 495, 64%), validation (n = 124, 16%), and test (n = 155, 20%) sets, corresponding to an overall (8:2):2 split.

For feature selection, variables with p < 0.05 in univariate analysis were retained as candidate predictors for multivariate modeling. Although methods such as decision tree and LASSO regression were explored, they did not yield superior performance compared with the univariate filtering approach.

The complete data processing pipeline and parameter settings were documented to enhance reproducibility. The implementation details of the SSA algorithm, including its configuration and code, have been made publicly available in a GitHub repository: https://github.com/elarabao/SSA-Medical-Prediction.

### 2.3 Salp swarm optimization algorithm

The Salp Swarm Optimization (SSA) algorithm, inspired by the collective foraging behavior of salps in marine environments, was employed to optimize the hyperparameters of our predictive models (Mirjalili et al., 2017). This metaheuristic algorithm effectively balances exploration and exploitation during the optimization process, making it particularly suitable for high-dimensional medical datasets.

#### 2.3.1 Mathematical formulation

The SSA algorithm simulates the chain behavior of salps, where the population is divided into leaders and followers. The position of each salp in the d -dimensional search space represents a potential solution (i.e., a set of hyperparameters). The mathematical model consists of two main phases, which are defined by Equations 1–6.

1. Leader position update: The leader salp (best solution) guides the swarm toward the food source (optimal solution):

$$x_{j}^{1}=\left\{\begin{array}{cc} F_{j}+c_{1}\left(\left(ub_{j}-lb_{j}\right)c_{2}+lb_{j}\right)\; & \text{if }c_{3}<0.5\\ F_{j}-c_{1}\left(\left(ub_{j}-lb_{j}\right)c_{2}+lb_{j}\right)\; & \text{otherwise} \end{array}\right.$$ (1)

Where:

-
$x_{j}^{1}$ is the position of the leader in the $j^{th}$ dimension
-
$F_{j}$ is the position of the food source (current best solution)
-
$ub_{j}$ and $lb_{j}$ are the upper and lower bounds of the $j^{th}$ dimension
-
$c_{2},c_{3}$ are random numbers uniformly distributed in [0,1]
-
$c_{1}$ is the convergence control parameter:

$$c_{1}=2e^{-{\left(\frac{4t}{T}\right)}^{2}}$$ (2)

Where t is the current iteration and T is the maximum number of iterations. This adaptive parameter balances exploration (high $c_{1}$ values early in optimization) and exploitation (low $c_{1}$ values later in optimization).

2. Follower position update: Followers move in a chain-like formation based on their preceding neighbor:

$$x_{j}^{i}=\frac{1}{2}\left(x_{j}^{i}+x_{j}^{i-1}\right)$$ (3)

Where $x_{j}^{i}$ is the position of the $i^{th}$ follower salp in the $j^{th}$ dimension.

#### 2.3.2 Implementation in medical prediction models

The SSA algorithm was implemented to optimize two key predictive models:

- Random Forest Optimization: The algorithm searched for optimal values of four critical hyperparameters:

$$\text{Position}=\left[n_{\text{estimators}},\text{max\_depth},\text{min\_samples\_split},\text{min\_samples\_leaf}\right]$$ (4)

With search boundaries: $n_{\text{estimators}}\in \left[50,500\right]$ , max_depth∈[3,30] , min_samples_split∈[2,20] , min_samples_leaf∈[1,10] .

- XGBoost Optimization: The algorithm optimized five key parameters:

$$\begin{array}{ll} & \text{Position}=\left[n_{\text{estimators}},\text{max\_depth},\text{learning\_rate},\text{gamma},\right.\\ & \;\left.\text{× min\_child\_weight}\right] \end{array}$$ (5)

With search boundaries: $n_{\text{estimators}}\in \left[50,500\right]$ , max_depth∈[3,15] , learning_rate∈[0.01,0.3] , gamma∈[0,1] , min_child_weight∈[1,10] .

Prior to the SSA optimization, the hyperparameters were initialized using a uniform random distribution within their predefined search boundaries for each dimension of the search space. Specifically, the initial positions of all salps were generated by sampling uniformly between the lower and upper bounds of each hyperparameter, thereby ensuring sufficient diversity in the initial population.If prior knowledge was available, the leader salp could optionally be initialized with a predefined parameter vector; otherwise, it was initialized in the same manner as the other salps. This initialization strategy provided SSA with a broad and unbiased starting point, facilitating effective exploration during the early optimization stages.

#### 2.3.3 Fitness function

The optimization objective was to minimize classification error rate evaluated through 5-fold cross-validation:

$$\text{Fitness}=1-\frac{1}{K}\overset{K}{\underset{k=1}{\sum }}{\text{Accuracy}}_{k}$$ (6)

Where K=5 represents the number of cross-validation folds, and ${\text{Accuracy}}_{k}$ is the classification accuracy on the $k^{th}$ validation fold.

The optimization process was implemented in Python 3.9 using NumPy and Scikit-learn libraries, with parallel computation to enhance efficiency.

### 2.4 Model construction

Data augmentation was performed using the SMOTE (Synthetic Minority Over-sampling Technique) method. Subsequently, feature selection was conducted through P < 0.05. After inputting the last nine clinical feature variables, seven machine learning algorithms were applied for model construction. These algorithms include Extreme Gradient Boosting (XGBoost), Logistic Regression (LR), Random Forest (RF), Gaussian Naive Bayes (GNB), Support Vector Machine (SVM), and k-Nearest Neighbor (KNN) models. Use these algorithms to predict the incidence of preoperative aortic dissection rupture.

### 2.5 Statistical analysis

The Shapiro-Wilk test was employed to assess data normality. Normally distributed data are expressed as mean ± standard deviation, and inter-group comparisons were conducted using the t-test. For non-normally distributed data, results are presented as median (M) with interquartile range (P25, P75), and differences were analyzed using the Mann-Whitney U test. Categorical data are expressed as frequency (percentage), with group comparisons performed using the chi-square test or Fisher’s exact test, as appropriate. Statistical significance was defined as p < 0.05. Model performance evaluation serves to compare the generalization capabilities of classifiers. Within the context of disease risk prediction, accuracy and recall are prioritized over other evaluation metrics. The following five performance metrics were used to evaluate the models: the area under the receiver operating characteristic curve (AUC-ROC), accuracy, precision, specificity, recall, and the F1 score.

## 3 Results

### 3.1 Clinical features of the patients

This prospective cohort study enrolled 774 consecutive patients [median age: 54 years (IQR 46–64); 86.5% male], including 47 cases (6%) with preoperative aortic rupture within 24 h of admission. Table1 demonstrates significant between-group disparities in baseline characteristics between the rupture (n = 47) and non-rupture (n = 728) cohorts.

**TABLE 1:** Preoperative characteristics and laboratory findings of the total population.

Variable	Total (n = 774)	Non-rupture (n = 727)	Rupture (n = 47)	*P*-value
Demographics				
Age (years)	54.00 (46.00–64.00)	54.00 (46.00–64.00)	63.00 (53.50–73.50)	<0.001
Male (%)	605 (78.2%)	575 (79.1%)	30 (63.8%)	0.023
SBP (mmHg)	138.00 (121.75–157.00)	138.50 (122.00–157.75)	118.50 (80.00–129.75)	<0.001
DBP (mmHg)	78.00 (67.00–90.25)	78.50 (67.25–91.00)	61.50 (59.25–77.75)	0.015
MAP (mmHg)	99.15 (95.42–102.33)	99.15 (94.83–103.33)	76.00 (63.92–97.42)	<0.001
Hypertension	240 (31.00%)	236 (32.50%)	4 (8.50%)	0.001
LVEF (%)	65.55 (63.40–67.00)	65.57 (63.00–67.00)	65.49 (64.35–65.85)	0.414
BMI	25.00 (24.00–27.00)	25.00 (24.00–27.00)	25.10 (24.10–25.60)	0.499
Obesity	300 (38.80%)	295 (40.60%)	5 (10.60%)	<0.001
Medical history				
Marfan Syndrome	10 (1.30%)	10 (1.40%)	0 (0.00%)	0.886
Hypertension(history)	383 (49.50%)	363 (49.90%)	20 (42.60%)	0.407
Diabetes Mellitus	28 (3.60%)	25 (3.40%)	3 (6.40%)	0.519
Cerebrovascular	41 (5.30%)	36 (5.00%)	5 (10.60%)	0.177
Symptoms and comorbidities				
Aortic insufficiency	664 (85.80%)	617 (84.90%)	47 (100.00%)	0.008
Cold extremities	4 (0.50%)	0 (0.00%)	4 (8.50%)	<0.001
Hypoxemia	10 (1.30%)	10 (1.40%)	0 (0.00%)	0.886
Hematochezia/Abdominal pain	7 (0.90%)	7 (1.00%)	0 (0.00%)	1.000
Neurological symptoms	35 (4.50%)	31 (4.30%)	4 (8.50%)	0.319
Syncope	12 (1.60%)	7 (1.00%)	5 (10.60%)	<0.001
Laboratory Findings				
pH (AB)	7.37 (7.33–7.41)	7.38 (7.34–7.41)	7.30 (7.23–7.34)	<0.001
Lac (AB) (mmol/L)	9.00 (2.30–19.00)	9.00 (2.30–18.90)	22.00 (4.60–57.10)	<0.001
PaO_2_ (mmHg)	101.00 (80.21–133.05)	101.00 (80.41–132.00)	108.00 (77.90–148.00)	0.471
OI (mmHg)	299.81 (237.25–368.50)	300.14 (239.00–367.00)	293.00 (210.50–386.50)	0.402
Neutrophil%	0.87 (0.80–0.90)	0.87 (0.79–0.90)	0.87 (0.82–0.91)	0.507
WBC ( $\times 10^{9}$ /L)	13.04 (10.03–15.86)	12.87 (9.85–15.63)	15.44 (13.43–18.17)	<0.001
RBC ( $\times 10^{12}$ /L)	4.42 (4.02–4.78)	4.43 (4.04–4.80)	4.27 (3.88–4.55)	0.027
PLT ( $\times 10^{9}$ /L)	186.00 (150.00–225.00)	187.00 (151.50–227.00)	165.00 (125.00–201.50)	0.002
TC (mmol/L)	3.34 (0.50–4.55)	3.35 (0.51–4.55)	3.02 (0.39–4.53)	0.288
Cr ( μ mol/L)	69.90 (10.98–100.58)	69.20 (10.84–98.20)	92.15 (20.10–144.00)	0.002
Alb (g/L)	33.60 (4.04–38.49)	33.90 (4.05–38.60)	28.30 (3.89–33.90)	0.007
eGFR (mL/min/1.73m^2^)	80.96 (58.56–97.10)	82.38 (61.23–97.85)	52.52 (35.68–67.07)	<0.001
D-dimer (mg/L)	7.88 (2.97–24.73)	6.98 (2.81–22.01)	30.40 (14.41–57.42)	<0.001
ALT (U/L)	17.00 (3.76–34.10)	16.50 (3.56–32.70)	32.74 (10.81–109.66)	0.001
AST (U/L)	22.80 (12.16–36.60)	22.20 (11.90–33.95)	39.60 (20.68–159.82)	<0.001

SBP: Systolic Blood Pressure (mmHg); DBP: Diastolic Blood Pressure (mmHg); MAP: Mean Arterial Pressure (mmHg); LVEF: Left Ventricular Ejection Fraction (%); AB: Arterial Blood; Lac: Lactate; PaO_2_: Partial pressure of oxygen; OI: Oxygenation Index; WBC: White Blood Cells; RBC: Red Blood Cells; PLT: Platelets; TC: Total Cholesterol; Cr: Creatinine; Alb: Albumin; eGFR: Estimated Glomerular Filtration Rate; ALT: Alanine Aminotransferase; AST: Aspartate Aminotransferase.

Demographic analysis revealed older age in the rupture group versus non-rupture controls (median 63 vs. 53 years; P<0.001 ). Laboratory profiling demonstrated a more pronounced inflammatory and coagulopathic state in rupture cases, evidenced by elevated leukocyte counts (median 12.3 vs. $7.9\times 10^{9}$ /L; P<0.001 ), higher plasma D-dimer levels (median 7.8 vs. 2.3 μ g/mL; P<0.001 ), and increased arterial lactate concentrations (median 4.1 vs. 1.8 mmol/L; P<0.001 ). Metabolic disturbances manifested through significantly reduced glomerular filtration rates (median 58 vs. 82 mL/min/ $1.73\;{\text{m}}^{2}$ ; P<0.001 ) and elevated plasma creatinine levels (median 1.5 vs. 1.1 mg/dL; P=0.002 ).

Hemodynamic evaluation showed comparable systolic (median 118 vs. 124 mmHg; P=0.089 ) and diastolic blood pressures (median 68 vs. 72 mmHg; P=0.093 ) between groups, but significantly lower mean arterial pressure in rupture cases (median 78 vs. 84 mmHg; P=0.038 ). Imaging biomarkers indicated greater aortic regurgitation severity (grade ≥ 3: 41.3% vs. 22.1% ; P=0.004 ) and widened alveolar-arterial oxygen gradient (median 32 vs. 22 mmHg; P=0.001 ) in the rupture group. Pericardial effusion prevalence ( 34.8% vs. 32.8% ; P=0.751 ) and left ventricular ejection fraction (median 58% vs. 59% ; P=0.414 ) showed no statistical significance. Variables “periaortic hematoma” and “systolic hypertension” were excluded from analysis due to absence from the dataset.

### 3.2 Prediction models’ performance comparison

**FIGURE 2:**
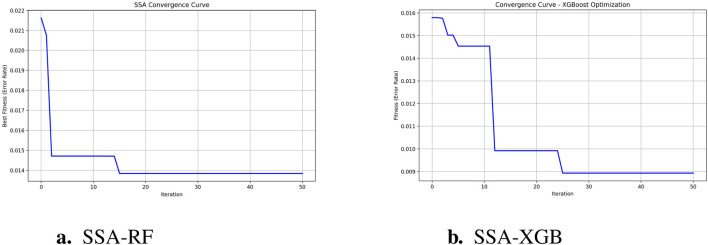
SSA-optimized model convergence curves. **(a)** SSA-RF Convergence (1 - accuracy); **(b)** SSA-XGBoostConvergence (1 - accuracy).

**FIGURE 3:**
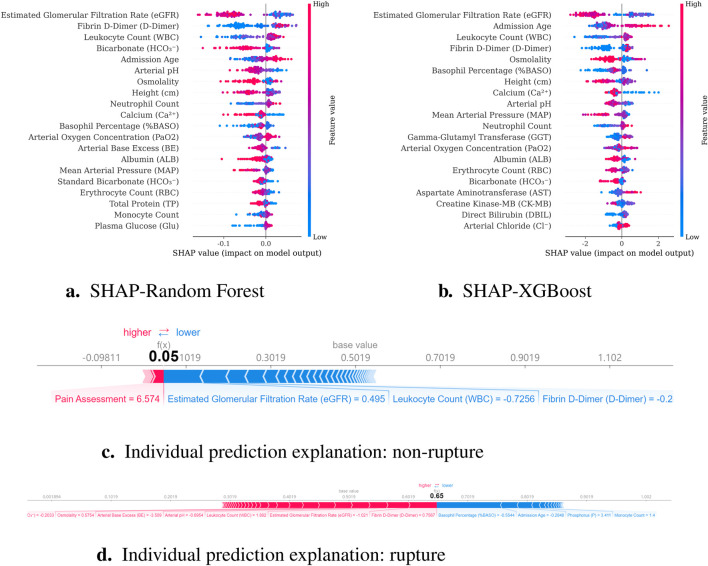
Model interpretability results combining global and local explanations. **(a)** SHAP summary for Random Forest; **(b)** SHAP summary for XGBoost; **(c)** Local SHAP force plot for a non-rupture patient; **(d)** Local SHAP force plot for a rupture patient.

To predict preoperative aortic dissection rupture within 24 h of admission, we evaluated seven machine learning models: logistic regression, decision tree, random forest, XGBoost, support vector machine (SVM), k-nearest neighbors (KNN), and multilayer perceptron (MLP). Key performance metrics are detailed in Table 2. Figure 2 shows the convergence patterns of the Salp Swarm Optimization–enhanced models, confirming their stable learning behavior. Figure 3 presents a comprehensive view of model interpretability. Global explanations from SHAP summary plots are provided for the Random Forest and XGBoost models (subfigures A and B), demonstrating the overall feature contributions across the dataset. Subfigures C and D further depict local SHAP force plots for individual patients, clearly highlighting the positive and negative feature influences in preoperative rupture prediction. In addition, These interpretability results provide robust evidence of the proposed framework’s clinical relevance and transparency, as detailed in the discussion.

**TABLE 2:** Model performance comparison.

Model	Accuracy	AUC	Precision	Recall	F1-score
Random Forest (Baseline)	0.94	0.95	0.53	0.80	0.64
Random Forest (SSA-Optimized)	0.97	0.98	0.75	0.90	0.82
XGBoost (Baseline)	0.93	0.94	0.45	0.50	0.47
XGBoost (SSA-Optimized)	0.94	0.94	0.50	0.70	0.58
Support Vector Machine	0.82	0.82	0.23	0.83	0.36
Logistic Regression	0.79	0.89	0.22	0.90	0.35
Decision Tree	0.88	0.70	0.26	0.50	0.34
K-Nearest Neighbors	0.75	0.80	0.15	0.60	0.24
Multilayer Perceptron	0.91	0.80	0.36	0.50	0.42

The SSA-optimized Random Forest demonstrated superior overall performance, achieving high accuracy (0.97) and the highest AUC (0.98) among all models. Crucially, it maintained balanced precision (0.75) and recall (0.90), yielding the optimal F1-score (0.82). Compared to its baseline version, SSA optimization yielded a 3% accuracy improvement (0.94 → 0.97) and 3% AUC enhancement (0.95 → 0.98) while preserving critical clinical balance between sensitivity and specificity. Similarly, SSA optimization moderately enhanced XGBoost performance, increasing accuracy by 1% (0.93 → 0.94) and maintaining AUC (0.94), while improving precision by 5 percentage points (0.45 → 0.50). This optimization elevated its F1-score from 0.47 to 0.58, representing a clinically significant 23% relative improvement in overall performance.

**FIGURE 4:**
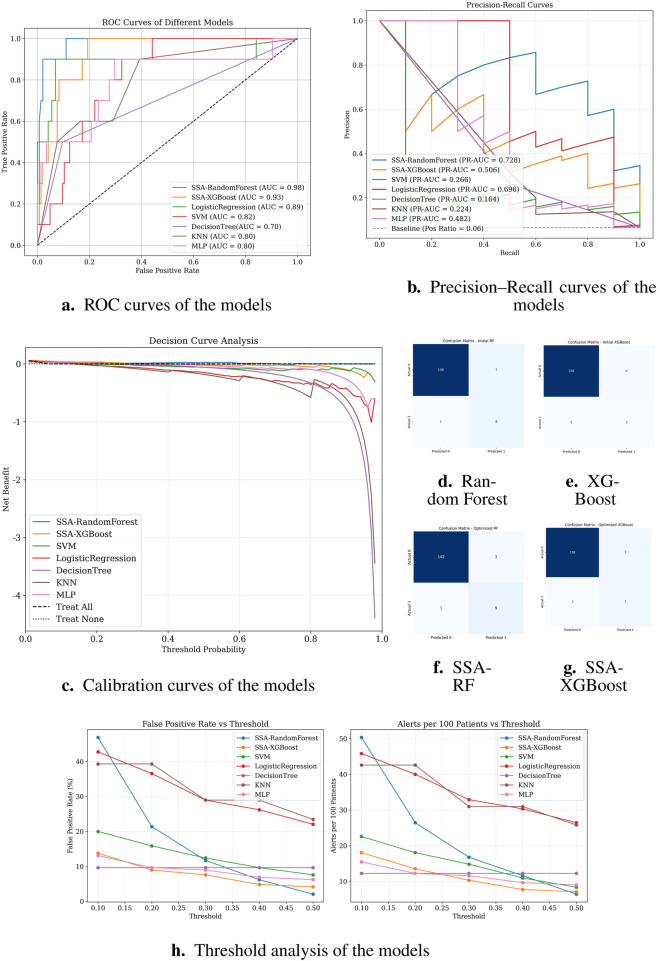
Classification performance of different models. **(a)** ROC curves; **(b)** Precision–Recall curves; **(c)** Calibration curves; **(d–g)** Confusion matrices of Random Forest, XGBoost, SSA-RF, and SSA-XGBoost models, respectively; **(h)** Threshold analysis plot.

Figure 4 demonstrates the classification performance of the proposed models. Subfigure (A) shows the receiver operating characteristic (ROC) curve, highlighting the superior discrimination ability of the SSA-optimized Random Forest model with an AUC of 0.98. Subfigures (D) through (G) display the confusion matrices of the Random Forest, XGBoost, SSA-RF, and SSA-XGBoost models, respectively. As shown, the SSA-enhanced models achieved higher true positive rates with fewer false negatives, reflecting improved sensitivity in predicting preoperative aortic rupture. These findings confirm that the proposed optimization strategy enhances model stability and accuracy, providing a clinically valuable tool for risk stratification in acute type A aortic dissection.

Notably, logistic regression demonstrated high sensitivity (recall = 0.90), theoretically identifying most potential rupture cases. However, its alarmingly low precision (0.22) implies that 78% of predicted high-risk patients would be false positives. In clinical practice, such over-alerting could lead to unnecessary invasive monitoring, increased healthcare costs, and patient distress, particularly in resource-constrained settings. Similarly, SVM showed high recall (0.83) but critically low precision (0.23), suggesting that only 23% of its positive predictions would be clinically valid. While decision tree models displayed moderate accuracy (0.88), their poor discriminative capacity (AUC = 0.70) and low precision (0.26) reflect inherent instability in handling complex medical data patterns.

Subfigure (B) presents the precision-recall (PR) curves. SSA-RandomForest achieved the highest PR-AUC (0.728), outperforming other models by maintaining both high recall and precision, which is crucial in minimizing false negatives without excessively increasing false positives. Logistic regression performed moderately well (PR-AUC = 0.696), whereas SSA-XGBoost showed modest improvement (PR-AUC = 0.506). In contrast, conventional models such as SVM, KNN, and decision trees exhibited markedly lower PR-AUC values, indicating limited robustness in imbalanced clinical data scenarios.

Subfigure (C) shows the decision curve analysis (DCA). SSA-RandomForest provided the greatest net clinical benefit across a wide range of threshold probabilities, consistently outperforming alternative models and the “treat all” or “treat none” strategies. SSA-XGBoost also demonstrated modest net benefit, whereas logistic regression, SVM, and other baseline classifiers contributed little or no clinical utility. These results underscore the superior clinical applicability of SSA-RandomForest, supporting its use in preoperative rupture risk prediction for ATAAD patients.

Notably, logistic regression demonstrated high sensitivity (recall = 0.90), theoretically identifying most potential rupture cases. However, its alarmingly low precision (0.22) implies that 78% of predicted high-risk patients would be false positives. In clinical practice, such over-alerting could lead to unnecessary invasive monitoring, increased healthcare costs, and patient distress, particularly in resource-constrained settings. Similarly, SVM showed high recall (0.83) but critically low precision (0.23), suggesting that only 23% of its positive predictions would be clinically valid. While decision tree models displayed moderate accuracy (0.88), their poor discriminative capacity (AUC = 0.70) and low precision (0.26) reflect inherent instability in handling complex medical data patterns.

These findings collectively suggest that random forest optimally balances predictive power and clinical utility, minimizing both missed diagnoses and unnecessary interventions.

## 4 Discussion

Acute type A aortic dissection (ATAAD) remains a life-threatening cardiovascular emergency characterized by abrupt onset, rapid progression, and prohibitive mortality rates (Larsson et al., 2024). While emergent surgical intervention represents the cornerstone of therapeutic management Chen et al. (2019), its implementation necessitates not only specialized vascular surgical expertise but also coordinated multidisciplinary support and advanced critical care infrastructure. These stringent requirements have driven regional centralization of tertiary care facilities, creating complex resource allocation challenges in high-volume centers. Whereas the majority of prior machine-learning studies in aortic dissection, exemplified by Wen et al.’s multicenter SHAP-interpretable model for postoperative reintubation Wen et al. (2025), have emphasized postoperative risk prediction, our work addresses the complementary yet distinct challenge of preoperative rupture risk stratification in ATAAD. The machine learning-based prognostic model developed herein integrates multidimensional clinical parameters through ensemble algorithms, offering clinicians a data-driven framework for surgical prioritization. By stratifying rupture risk profiles, this tool facilitates optimized resource utilization that may significantly reduce ATAAD-related mortality. The clinical utility extends beyond individual decision-making, as implementation of such predictive systems enables more efficient allocation of scarce resources in overloaded tertiary referral centers.

Building upon established associations between renal dysfunction and adverse outcomes, our study provides novel mechanistic insights into the renal-aortic interplay through SHAP interpretability analysis, identifying reduced estimated glomerular filtration rate (eGFR) as a critical biomarker for preoperative rupture risk in aortic dissection. This finding aligns with Jin et al. (2025) and is pathophysiologically supported by a dual-pathway framework: (1) Chronic poorly controlled hypertension induces renal arteriolosclerosis and parenchymal damage, exacerbating vascular wall degeneration to elevate rupture risk Zhang X. et al. (2021); (2) Concurrently, dissection extension into renal arteries establishes false lumen-dominant perfusion, causing renal ischemia and elevated false lumen pressure that promotes rupture (Jin et al., 2025). The SHAP analysis further highlighted white blood cell count (WBC) as a significant preoperative rupture predictor, with elevated levels demonstrating dose-dependent risk association. This finding aligns with existing literature where Chen et al. (2017) reported 26.1% in-hospital mortality during circulatory arrest in patients with WBC $<11\times 10^{9}$ /L, suggesting paradoxical immunomodulatory effects in acute aortic syndromes. Complementing this, Zhang et al. (2021a) identified sustained WBC elevation as a consistent mortality predictor across multiple postoperative timepoints. While conventional wisdom associates leukocytosis with inflammatory complications, our analysis suggests WBC may serve as a systemic stress biomarker reflecting aortic wall instability rather than secondary infection. Contrasting with established postoperative hypertension correlations Luehr et al. (2023), our model revealed preoperative hypotension as a critical rupture predictor. This hemodynamic paradox finds clinical resolution through recognition of two distinct pathophysiological states: First, Li et al. (2017) identified systemic hypotension as a marker of impending circulatory collapse rather than therapeutic success, while Dong et al. (2023) demonstrated 82% prevalence of preoperative shock in rupture cases. Expanding on these clinical observations, we hypothesize that acute blood pressure reduction in type A dissection may specifically correlate with impending pericardial compromise. When dissection propagates into the pericardial cavity, rapid hematoma accumulation can induce cardiac tamponade physiology. This process creates a biphasic hemodynamic response: initial compensatory tachycardia maintains cardiac output during early diastolic impairment, followed by abrupt decompensation as ventricular filling becomes restricted.

Notably, Dong et al. (2023) cohort revealed that 76% of patients with preoperative rupture exhibited electrocardiographic evidence of cardiac tamponade prior to circulatory collapse. This anatomical consideration reconciles the apparent contradiction between systemic hypotension and ongoing dissection propagation. Rather than reflecting therapeutic success, a sudden blood pressure drop in this context likely represents the transition from compensated shock to irreversible cardiovascular failure. Such hemodynamic instability necessitates urgent surgical intervention, as medical stabilization strategies may paradoxically delay definitive repair in patients with evolving tamponade. These findings underscore the critical distinction between therapeutic blood pressure control and pathologic hypotension resulting from progressive hemodynamic decompensation. The negative correlation with blood pressure parameters in our model thus serves as a vital prognostic indicator, necessitating careful clinical contextualization when interpreting hemodynamic trends during preoperative management.

Among the evaluated machine learning models, the random forest (RF) algorithm exhibited the most robust overall performance in predicting the rupture of ATTAD, achieving balanced metrics in both recall and precision. Following the integration of the Salp Swarm Algorithm (SSA) for hyperparameter optimization, the RF model further improved in accuracy and AUC while maintaining stable precision and recall. This suggests that SSA can efficiently fine-tune the ensemble’s decision boundaries, helping to reinforce generalizability without compromising interpretability (Mirjalili et al., 2017). The SSA-optimized RF model thus combines strong baseline performance with refined parameter calibration, reinforcing its suitability for medical predictive tasks. Similarly, the application of SSA to XGBoost yielded substantial gains in precision, improving from 0.56 to 0.71, while maintaining high recall. This improvement indicates that SSA effectively enhances XGBoost’s capability to discriminate true positive cases from false positives, an essential aspect for clinical risk stratification. The superior adaptability of tree-based algorithms to SSA-driven optimization likely stems from their sensitivity to hyperparameters, which benefit from SSA’s dynamic exploration of the search space. The logistic regression (LR) model demonstrated competitive recall despite its relatively lower precision. This phenomenon aligns with theoretical expectations in imbalanced datasets where the minority class is underrepresented. Under such conditions, models like logistic regression tend to prioritize sensitivity at the expense of precision – a trade-off that may be clinically acceptable, or even strategically preferable, in screening contexts where missing true positive cases could have severe consequences. Consequently, while exhibiting suboptimal precision, the logistic regression model retains clinical utility as a conservative screening instrument within multi-stage diagnostic protocols.

Taken together, these results highlight a complementary diagnostic strategy: LR could serve as a high-sensitivity frontline screening tool to minimize false negatives, with subsequent confirmation by SSA-optimized RF or XGBoost models to improve specificity and reduce unnecessary interventions. The current predictive model was developed based on structured clinical parameters, as standardized CTA image feature extraction remains unavailable in our ongoing specialty database. Furthermore, this single-center retrospective analysis may limit generalizability due to regional patient demographics and clinical practice patterns. Multicenter prospective validation incorporating advanced imaging features is planned to strengthen its clinical utility.

## Data Availability

The raw data supporting the conclusions of this article will be made available by the authors, without undue reservation.
